# LEFTY2 inhibits endometrial receptivity by downregulating Orai1 expression and store-operated Ca^2+^ entry

**DOI:** 10.1007/s00109-017-1610-9

**Published:** 2017-12-11

**Authors:** Madhuri S. Salker, Yogesh Singh, Ruban R. Peter Durairaj, Jing Yan, Md Alauddin, Ni Zeng, Jennifer H. Steel, Shaqiu Zhang, Jaya Nautiyal, Zoe Webster, Sara Y. Brucker, Diethelm Wallwiener, B. Anne Croy, Jan J. Brosens, Florian Lang

**Affiliations:** 1 Department of Women’s Health, Eberhard-Karls University Tuebingen, Calwerstr 7, D-72076 Tuebingen, Germany; 2 Department of Medical Genetics and Applied Genomics, Eberhard-Karls University Tuebingen, Calwerstr 7, D-72076 Tübingen, Germany; 3 Division of Biomedical Sciences, Warwick Medical School, Coventry, CV2 2DX UK; 4 Department of Physiology I, Eberhard-Karls University Tuebingen, Wilhelmstr 56, D-72074 Tuebingen, Germany; 5 Department of Surgery and Cancer, Institute of Reproductive and Developmental Biology, Faculty of Medicine, Imperial College London, London, W12 0NN UK; 6 ES Cell and Transgenics Facility, Medical Research Council Clinical Sciences Centre, Imperial College London, Du Cane Road, London, W12 0NN UK; 7 Department of Biomedical and Molecular Sciences, Queen’s University, Kingston, Ontario Canada; 8 Tommy’s National Centre for Miscarriage Research, University Hospitals Coventry and Warwickshire NHS Trust, Clifford Bridge Rd, Coventry, CV2 2DX UK; 9 Department of Molecular Medicine II, Heinrich Heine University Düsseldorf, Universitätsstr 1, D-40225 Düsseldorf, Germany

**Keywords:** Implantation, LEFTY2, ORAI1, SOCE, Calcium, Pregnancy, Miscarriage

## Abstract

**Abstract:**

Early embryo development and endometrial differentiation are initially independent processes, and synchronization, imposed by a limited window of implantation, is critical for reproductive success. A putative negative regulator of endometrial receptivity is LEFTY2, a member of the transforming growth factor (TGF)-β family. LEFTY2 is highly expressed in decidualizing human endometrial stromal cells (HESCs) during the late luteal phase of the menstrual cycle, coinciding with the closure of the window of implantation. Here, we show that flushing of the uterine lumen in mice with recombinant LEFTY2 inhibits the expression of key receptivity genes, including *Cox2*, *Bmp2*, and *Wnt4*, and blocks embryo implantation. In Ishikawa cells, a human endometrial epithelial cell line, LEFTY2 downregulated the expression of calcium release-activated calcium channel protein 1, encoded by *ORAI1*, and inhibited store-operated Ca^2+^ entry (SOCE). Furthermore, LEFTY2 and the Orai1 blockers 2-APB, MRS-1845, as well as YM-58483, inhibited, whereas the Ca^2+^ ionophore, ionomycin, strongly upregulated *COX2*, *BMP2* and *WNT4* expression in decidualizing HESCs. These findings suggest that LEFTY2 closes the implantation window, at least in part, by downregulating Orai1, which in turn limits SOCE and antagonizes expression of Ca^2+^-sensitive receptivity genes.

**Key messages:**

•Endometrial receptivity is negatively regulated by LEFTY2.

•LEFTY2 inhibits the expression of key murine receptivity genes, including *Cox2*, *Bmp2* *and Wnt4*, and blocks embryo implantation.

•LEFTY2 downregulates the expression of Orai1 and inhibits SOCE.

•LEFTY2 and the Orai1 blockers 2-APB, MRS-1845, and YM-58483 inhibit *COX2*, *BMP2*, and *WNT4* expression in endometrial cells.

•Targeting LEFTY2 and Orai1 may represent a novel approach for treating unexplained infertility.

**Electronic supplementary material:**

The online version of this article (10.1007/s00109-017-1610-9) contains supplementary material, which is available to authorized users.

## Introduction

A transient window of uterine receptivity ensures that embryos implant in an optimal endometrial environment [1]. Failure to establish [2] or premature closure [1, 3, 4] of the implantation window is thought to be a major cause of infertility, which affects ~ 15% of couples. Conversely, prolonged receptivity may lead to out-of-phase implantation and miscarriage [3, 4] or recurrent pregnancy loss [1, 4, 5]. The stromal signals responsible for terminating the window of implantation are not well characterized. A putative candidate is Left-Right Determination Factor 2 (LEFTY2), a cytokine highly expressed by decidualizing stromal cells during the late secretory phase of the menstrual cycle [6], i.e., following closure of the window of implantation and prior to menstruation [7]. LEFTY2, initially designated endometrial bleeding associated factor (EBAF), is a secreted ligand of the transforming growth factor-beta (β) superfamily of proteins [7, 8]. Following proteolytic processing of the secreted precursor, LEFTY2 acts as an antagonist of the Nodal signaling pathway by interfering both with the binding of NODAL to the activin receptor and the formation of a receptor complex [9]. During the late luteal phase of the menstrual cycle, strong LEFTY2 immunoreactivity was found in the stroma and to a lesser extent in the endometrial glands [10]. Furthermore, LEFTY2 has also been detected in the endometrial fluid of fertile women, indicating that LEFTY2 is secreted into the lumen of the uterus. Interestingly, induction of the proprotein convertase (PC) 5/6, which processes LEFTY2, is triggered in the mouse uterus in response to artificial decidualization with oil or upon mechanical injury [11]. Endometrial LEFTY2 is elevated during the receptive phase in some patients with “unexplained infertility” suggesting that dysregulation of LEFTY2 contributes to infertility [12, 13]. Further, in vivo gene transfer of *Lefty2* in the mouse uterus leads to implantation failure [14]. However, the mechanisms underlying the negative impact of LEFTY2 on endometrial receptivity remain largely unknown.

Several implantation events, including blastocyst-endometrium adhesion [15], regulation of growth factor signaling [16], transcription factor activity [17], epithelial tight junctions [18], protease activity [19], cyclooxygenase 2 (COX2)-dependent prostaglandin production [20, 21], and epithelial transport [22, 23] are regulated by Ca^2+^ signaling. Embryo-derived tryptic serine proteases have emerged as important implantation signals, capable of inducing COX2-dependent prostaglandin E_2_ (PGE_2_) production in response to cytosolic Ca^2+^([Ca^2+^]_i_) oscillations [21]. [Ca^2+^]_i_ is tightly regulated by several mechanisms, including Ca^2+^ release from intracellular stores and subsequent activation of store-operated Ca^2+^ entry (SOCE) [24]. SOCE is initiated by the Ca^2+^ sensor proteins, stromal interaction molecule 1 (STIM1) and STIM2, that are located within the endoplasmic reticulum (ER) [25]. Following store depletion, STIMs cluster and trap the plasma membrane (PM) proteins ORAI1–3 into ER-PM junctions [24]. These regions become sites of highly selective Ca^2+^ entry, predominantly through ORAI assembled channels [26, 27]. Prolonged and disordered Ca^2+^ oscillations in decidualizing stromal cells in response to serine proteases secreted by low-quality human embryos are thought to trigger ER- stress, which in turn facilitates early maternal rejection of a non-viable conceptus [28]. Recently, we reported that Orai1 is expressed in the secretory phase endometrium as well as in endometrial carcinoma cells [29]. Both Orai1 expression and function are upregulated by TGFß1, an effect presumably participating in the regulation of endometrial regeneration [29]. The role of Orai1 in implantation remains to be determined.

The mechanisms regulating [Ca^2+^]_i_ levels in endometrial cells remain incompletely understood but presumably involve Ca^2+^ release from intracellular stores with subsequent activation of SOCE [27, 30–34]. Here, we show that LEFTY2 inhibits the expression of Ca^2+^ responsive receptivity genes by downregulating Orai1 expression and limiting SOCE. Our findings provide new insights into the molecular mechanisms that define a limited window of implantation in the human endometrium.

## Results

**Fig. 1:**
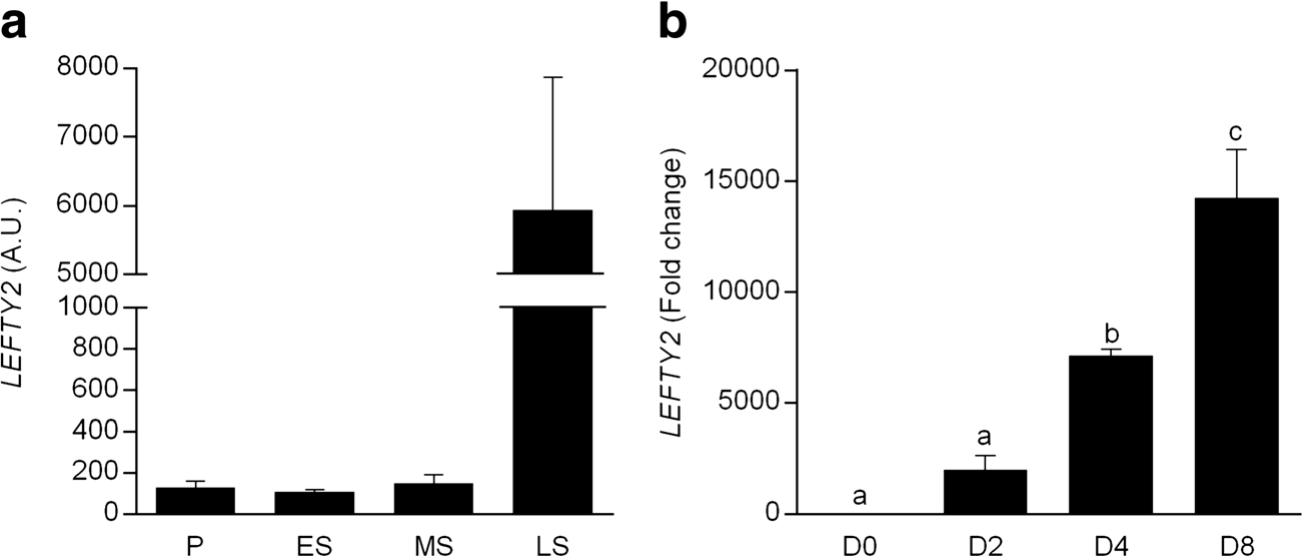
Expression of *LEFTY2* in human endometrium. **a** Arithmetic means ± SEM of *LEFTY2* transcripts expressed in arbitrary units (a.u.) in whole endometrial biopsies throughout the menstrual cycle. Expression levels were derived from in silico analysis of publicly available microarray data (GEO profile LEFTY2: ID 24460960). The phases of the cycle are indicated as follows: *P* proliferative, *ES* early-secretory, *MS* mid secretory, and *LS* late secretory. **b** Induction of *LEFTY2* in decidualizing HESCs. Arithmetic means ± SEM (*n* = 3) of transcript levels of *LEFTY2* measured in undifferentiated HESCs and cells decidualized with 8-br-cAMP and MPA for 2, 4 or 8 days. The data are expressed relative to that in undifferentiated cells (D0). Different letters above the error bars indicate that those groups are significantly different from each other at *P < 0.05 using the Kruskal-Wallis test

Mining of publicly available microarray data (Gene Expression Omnibus accession number: 24460960) demonstrated a marked increase (30-fold) in *LEFTY2* mRNA levels in whole endometrial biopsies upon transition from mid-secretory (MS; receptive) to late secretory (LS; refractory) phase of the menstrual cycle (Fig. 1a). In culture, decidual transformation of HESCs increased *LEFTY2* expression. After 8 days of differentiation, *LEFTY2* mRNA levels increased approximately 14,000-fold (Fig. 1b).

**Fig. 2:**
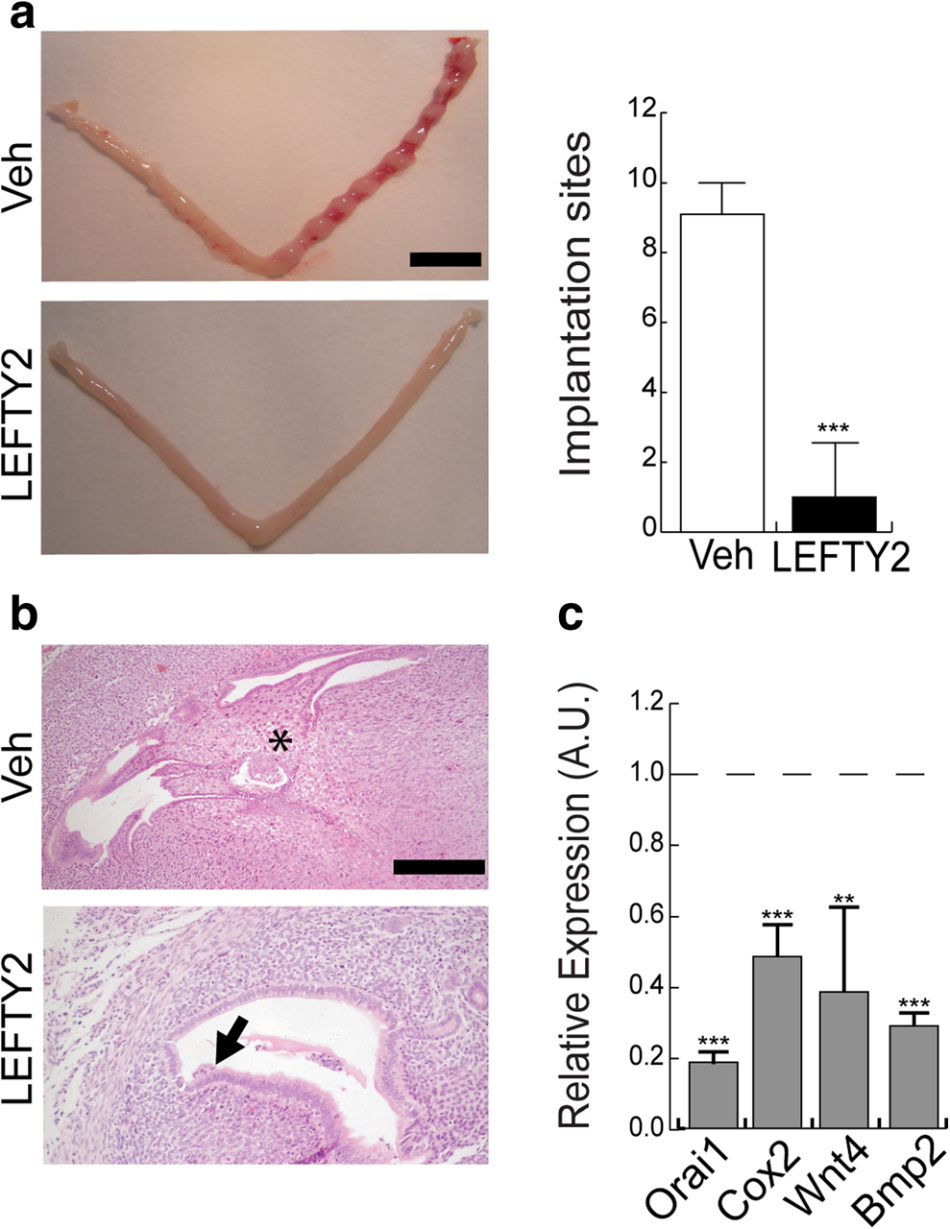
Recombinant LEFTY2 blocks embryo implantation. Vas mated C57BL/6 female mice were subjected to laparotomy and both uterine horns gently flushed with either vehicle (PBS) or recombinant LEFTY2 (500 ng/ml). Ten embryos (equivalent to stage 3.5 dpc) were then transferred to the right horn. Each treatment group consisted of five mice. **a** The gross morphological appearance of the uteri and implantation sites at 9.5 dpc (scale bar, 1 cm); the right panel shows arithmetic means ± SEM (*n* = 5) of the number of implantation sites following uterine flushing with PBS (Veh) or recombinant LEFTY2. **b** Corresponding histological appearance of implantation sites. The upper panel (Veh; control) shows a normal blastocyst. The lower panel shows fetal demise following uterine LEFTY2 exposure prior to embryo transfer (scale bar, 100 μm). **c** Arithmetic means ± SEM (*n* = 5) of the relative expression of murine *Orai1*, *Cox2 Wnt4* and *Bmp2*, transcript levels, normalized to *Cyclo *(housekeeping) mRNA and expressed in arbitrary units (a.u.), following uterine flushing with LEFTY2 or vehicle (PBS, dotted line). **P < 0.01 and ***P < 0.001 indicate statistically significant difference to absence of LEFTY2 (Student’s *t* test)

In order to explore the functional significance of increased LEFTY2 expression, we examined the effect of LEFTY2 on embryo implantation in the mouse uterus. Embryo implantation in mice is a stepwise process that starts with attachment of the blastocysts to the endometrial luminal epithelial layer between 3.5 to 4.5 days postcoitus (dpc), triggering decidualization of the underlying stromal cells [35] and closure of the uterine lumen [36].To confirm that LEFTY2 renders the endometrium refractory to implantation [14], murine uterine flushing experiments were performed in C57BL/6 female mice mated with vasectomized males. The uterine lumen of pseudopregnant female mice was injected with recombinant LEFTY2 before transfer of 10 cultured blastocysts equivalent of 3.5 dpc into a single uterine horn. Phosphate-buffered saline (PBS; vehicle) was flushed through the uterus prior to embryo transfer in control animals. The number of implantation sites was determined 72 h later. Out of 50 embryos transferred in each group, 43 (86%) embryos failed to implant in uteri treated with recombinant LEFTY2. By contrast, only 5 (10%) embryos failed to implant following flushing of the uterine lumen with PBS (Fig. 2a). Viable embryos were observed in the implantation sites of control (Veh) animals (Fig. 2b). The few established implantation sites following LEFTY2 exposure appeared smaller and the presence of collapsed blastocysts suggested imminent demise (Fig. 2b). To exclude a possible effect of LEFTY2 on the blastocysts, we performed further flushing experiments. C57BL/6 female mice were mated with fertile males to induce a normal pregnancy. At day 3.0 dpc, the uterus was flushed once either with PBS or LEFTY2. In this natural mating model, the blastocysts are still within the oviduct at day 3.0 dpc and are therefore not exposed to recombinant LEFTY2. Our results show again a significant decrease of implantation sites in LEFTY2 flushed mice (Supplementary Fig. 1), further suggesting that LEFTY2 blocks implantation by acting directly on the endometrium.

We speculated that failed implantation following LEFTY2 exposure could reflect inhibition of key murine implantation genes. In agreement, murine levels of *Orai1*, *Bmp2*, *Cox2* and *Wnt4* were significantly downregulated in LEFTY2-treated uteri when compared to control mice (Fig. 2c). Other cardinal murine implantation genes, including *Lif*, *Ihh*, *Hoxa10* and *Hbegf* were unaffected by LEFTY2 (Supplementary Fig. 2).

**Fig. 3:**
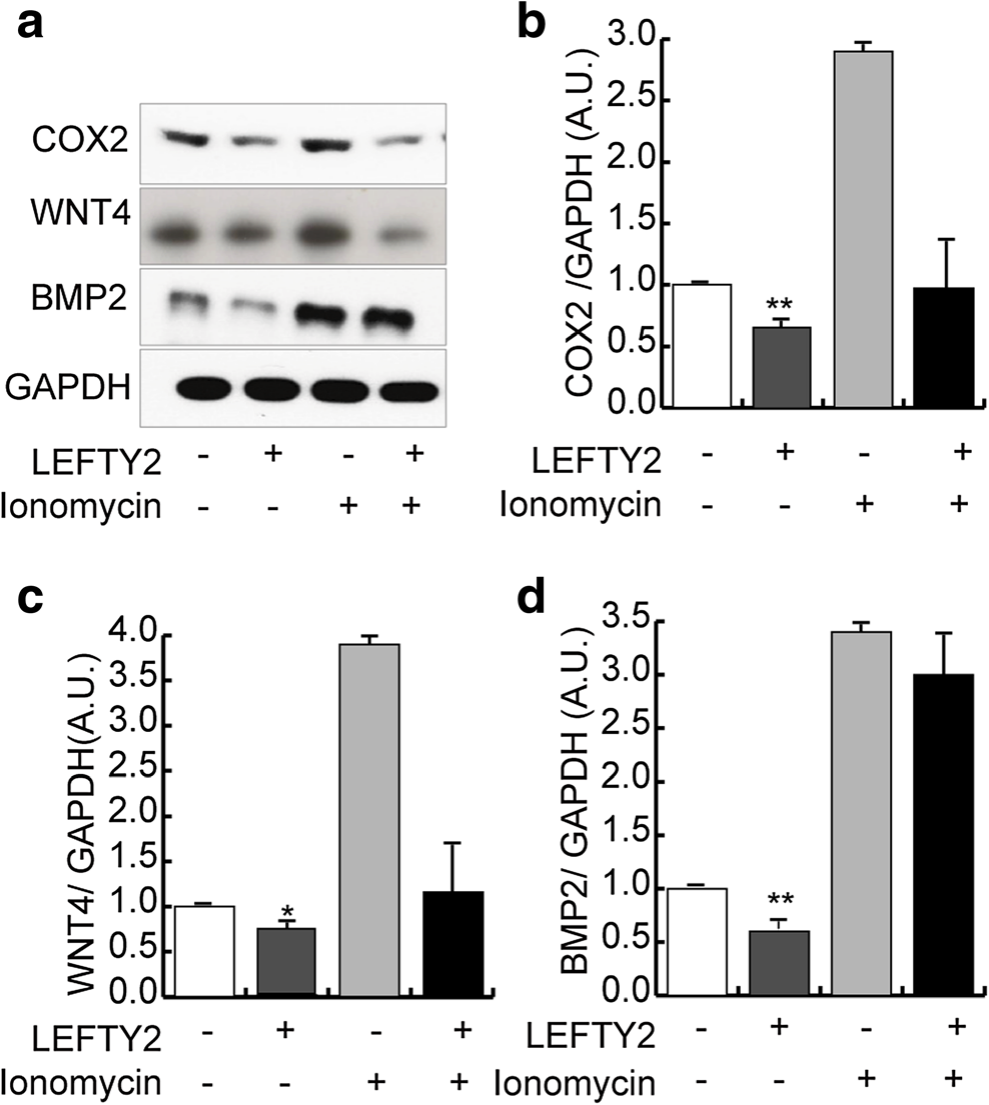
Differential regulation of Ca^2+^-dependent receptivity factors in response to LEFTY2 and Ionomycin. Primary HESC cultures were treated with 8-Br-cAMP and MPA for 6 days with or without LEFTY2 (25 ng/ml) or Ionomycin (1 μM) or in combination. **a** Original Western blots showing COX2, WNT4, BMP2 and GAPDH. **b** *–* **d** The abundance of COX2, WNT4 and BMP2 was quantified by ImageJ analysis of six Western blots and normalized to GAPDH. The data shows arithmetic means ± SEM. *P < 0.05, **P < 0.01 indicate significant difference to absence of LEFTY2 (Student’s *t* test)

To determine the relevance of these observations to the human endometrium, we decidualized HESCs with 8-Br-cAMP and MPA (medroxyprogesterone acetate, a progestin) for 6 days in the presence or absence of LEFTY2. As was the case in mice, recombinant LEFTY2 also attenuated the expression of WNT4*,* BMP2, and COX2 at protein and mRNA level in decidualizing HESCs (Fig. 3 and Supplementary Fig. 3). By contrast, endometrial WNT4*,* BMP2 and COX2 expression was markedly upregulated in response to the Ca^2+^ ionophore, ionomycin (Fig. 3), indicating that the expression of these implantation factors is responsive to increase in [Ca^2+^]_i_. Interestingly, LEFTY2 abrogated the induction of COX2 and WNT4 in response to Ionomycin, although it failed to reverse the induction of BMP2. Ionomycin treatment had no impact on *LIF*, *IHH*, *HOXA10*, or *HBEGF* mRNA levels in decidualizing HESCs (Supplementary Fig. 4).

**Fig. 4:**
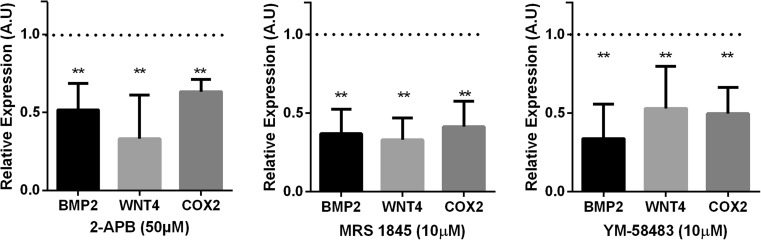
Orai1 inhibitors 2-APB, MRS-1845, and YM-58483 inhibit Ca^2+^-responsive implantation genes. Arithmetic means ± SEM (*n* = 6) of *BMP2*, *WNT4*, and *COX2* transcript levels relative to that of *L19* (housekeeping) in primary HESC cultures that were treated with 8-Br-cAMP and MPA for 6 days in the absence (dotted line) or in presence of the Orai-1 inhibitors 2-APB (50 μM), MRS-1845 (10 μM), or YM-58483 (10 μM); **P < 0.01 indicates significant difference from the control using Student’s *t* test

In order to determine if the inhibition of Ca^2+^-responsive receptivity genes following LEFTY2 treatment was a consequence of SOCE inactivation, primary HESCs were decidualized with 8-Br-cAMP and MPA for 6 days in the absence or presence of Orai1 inhibitors 2-aminoethoxydiphenyl borate (2-APB) [29], YM-58483 [37] or MRS-1845 [38]. Induction of *IGFBP1* and *PRL* transcript levels confirmed that the cells mounted a decidual response (data not shown). As shown in Fig. 4, exposure of decidualizing HESC Orai1 inhibitors 2-APB, YM-58483 and MRS-1845 significantly decreased *COX2*, *BMP2* and *WNT4* transcript levels.

**Fig. 5:**
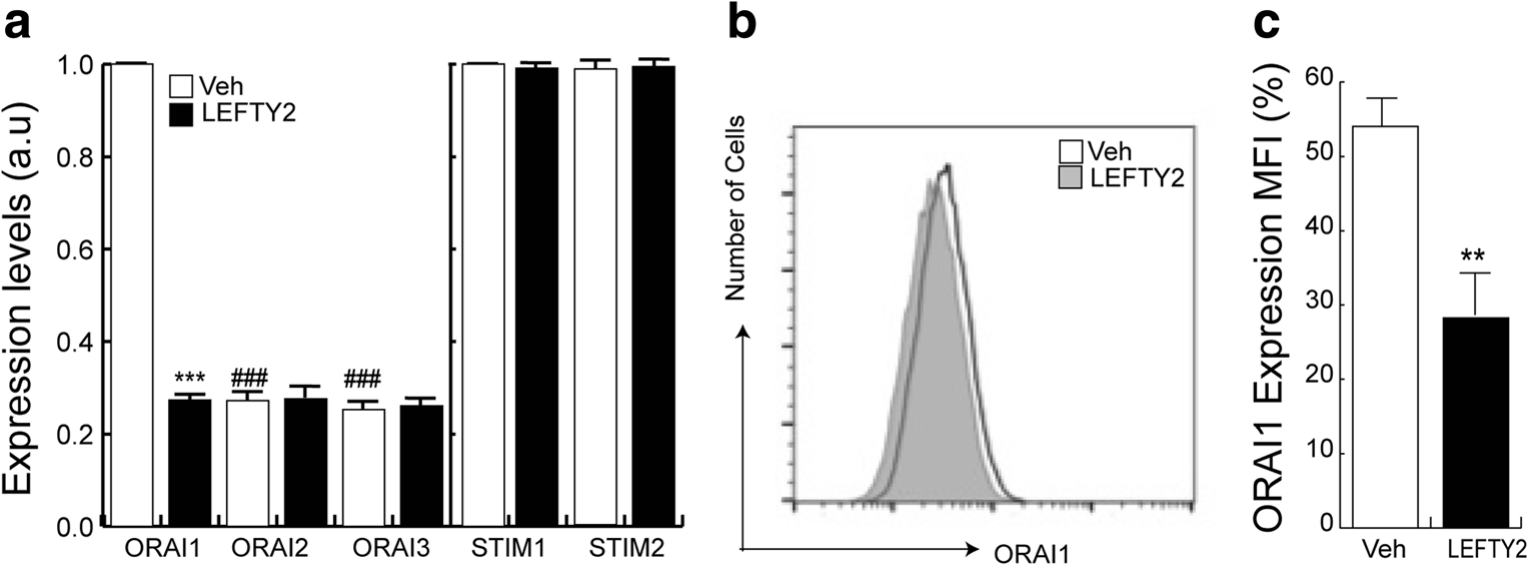
LEFTY2 downregulates endometrial Orai1 expression. **a** Endometrial Ishikawa cells were treated with or without LEFTY2 (25 ng/ml) for 6 days, and *ORAI1, ORAI2, ORAI3, STIM1* and *STIM2 mRNA* levels were measured. The results show arithmetic means ± SEM (*n* = 6). Expression levels of the *ORAI2* and *ORAI3* isoforms are relative to the *ORAI1* transcript level in the absence of recombinant LEFTY2 treatment. **b** and **c** Original FACS plot and bar graph of ORAI1 expression in parallel cultures treated with and without LEFTY2. **P < 0.01, ***P < 0.001 indicates statistically significant difference to absence of LEFTY2. ^###^ *P* < 0.001 indicates statistically significant difference from Control-Orai1 transcripts (Student’s *t* test)

We hypothesized that LEFTY2 may similarly impair SOCE in luminal endometrial epithelial cells at implantation. To test this hypothesis, we first determined the transcript levels of the *ORAI1–3* and *STIM1–2* isoforms in endometrial Ishikawa cells treated with or without LEFTY2. Ishikawa cells are a commonly used cell line to study human implantation events [28, 39, 40]. As illustrated in Fig. 5a, LEFTY2 selectively decreased the transcript levels of *ORAI1*, the dominant *ORAI* isoform in these cells. The transcript levels for *ORAI2-3* were low in Ishikawa cells, irrespective of LEFTY2 treatment. Similarly, *STIM1-2* expression did not change in response to LEFTY2 treatment. The decrease of *ORAI1* transcript levels following LEFTY2 treatment was paralleled by a decrease of ORAI1 at the protein level. Flow cytometry showed a significant reduction in ORAI1 in response to LEFTY2 treatment (Fig. 5b–c).

**Fig. 6:**
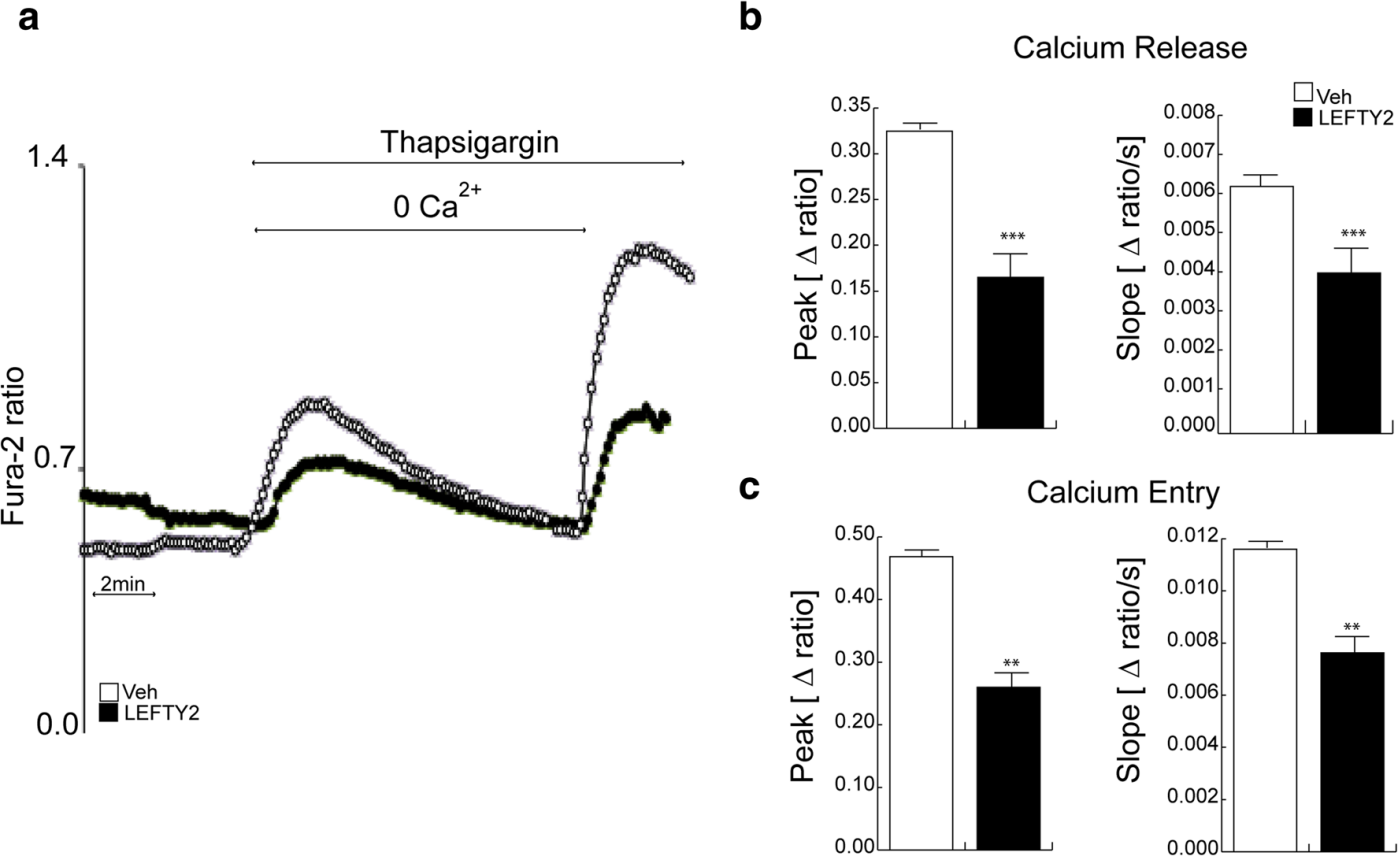
LEFTY2 reduces SOCE. **a** Original tracing of Fura-2 fluorescence-ratio in fluorescence spectrometry during and after Ca^2+^ depletion (1 μM thapsigargin) in LEFTY2 treated (black circles) and vehicle treated (PBS; white circles) Ishikawa cells. **b** *–* **c** Arithmetic means ± SEM (*n* = 6, each experiment 15–30 cells) of the peaks (left panels) and slopes (right panels) of **b** Ca^2+^ release and **c** Ca^2+^ entry, respectively **P < 0.01, ***P < 0.001 indicates significant difference from absence of LEFTY2 (Student’s *t* test)

In order to explore whether LEFTY2 impacts on Ca^2+^ signaling, Ishikawa cells were treated with thapsigargin, a potent inhibitor of the sarco/endoplasmic reticulum Ca^2+^ ATPase (SERCA), in the presence or absence of recombinant LEFTY2. Treatment with thapsigargin in the absence of extracellular Ca^2+^elicited a transient increase in [Ca^2+^]_i_(Δ[Ca^2+^]_i_) (Fig. 6a). Both peak and slope of Δ[Ca^2+^]_i_ were significantly blunted in response to LEFTY2 treatment. Subsequent addition of extracellular Ca^2+^sharply increased [Ca^2+^]_i_, reflecting SOCE. Peak and slope of Ca^2+^entry were significantly decreased by LEFTY2 (Fig. 6b–c). Basal Ca^2+^ levels were not significantly modified by LEFTY2 (Supplementary Fig. 5). Taken together, the data show that LEFTY2 blunts intracellular Ca^2+^release and SOCE.

## Discussion

LEFTY2, a member of the TGF-β superfamily, is implicated in embryo development and stem cell differentiation through its antagonistic action on the TGF-β/Smad signaling pathway [41–43]. LEFTY2 is also expressed in the human endometrium, most prominently during the late secretory phase of the menstrual cycle [10, 44, 45]. Previous studies reported that overexpression of LEFTY2 is associated with unexplained infertility [46] and irregular menstrual bleeding [8], and in vivo gene transfer studies in mice have also shown inhibition of implantation [14]. Furthermore, auto/paracrine LEFTY signaling attenuates decidual transformation of HESCs [47, 48], although the underlying mechanisms are incompletely understood.

In this study, we demonstrate that LEFTY2 renders the endometrium refractory to implantation, an effect paralleled by downregulation of ORAI1 expression and of SOCE in endometrial epithelial cells. By attenuating [Ca^2+^]_i_ signaling, LEFTY2 decreases expression of Ca^2+^-responsive implantation genes such as *COX2*, *WNT4* and *BMP2*. Other key implantation genes, including *LIF*, *IHH*, *HOXA10* and *HBEGF* transcripts [12, 49], were not affected by LEFTY2 or by Ionomycin treatment.

The pathways that couples LEFTY2 to transcriptional repression of *ORAI1* and the mechanisms linking ORAI1 function and increases of [Ca^2+^]_i_ to expression of receptivity genes requires further elucidation. The present observations uncover a novel function of Orai1 and store-operated Ca^2+^ entry. SOCE is known to trigger Ca^2+^ oscillations [27, 50], which stimulate several cellular functions [51–55] including entering into the S and the M phase of the cell cycle [56, 57] as well as cell survival [58, 59]. In contrast, sustained increase of cytosolic Ca^2+^ activity leads to apoptosis [53, 55, 60–68]. By mediating SOCE and oscillations of cytosolic Ca^2+^ activity, Orai1-3 and their regulators STIM 1–2 are decisive for cell survival [69–72]. It is noteworthy that ORAI1/STIM1 is upregulated by the serum and glucocorticoid inducible kinase (SGK1) and TGF-β in endometrial cancer cells [29], but downregulated by the AMP activated kinase (AMPK) [73]. Although as yet untested, downregulation of Orai1/STIM1 in response to AMPK activation potentially plays a role in preventing implantation of an embryo into an energy-deficient endometrium.

The present observations point to a new role of ORAI1-dependent SOCE in the regulation of endometrial receptivity genes, and hence the likelihood of successful embryo implantation. However, our findings do not rule out the involvement of additional mechanisms of Ca^2+^ release and/or entry during implantation. For example, evidence has emerged to implicate voltage-gated Ca^2+^ channels in Ca^2+^ entry in endometrial cells exposed to embryonic proteases [21]. Moreover, progesterone-dependent induction of phospholipase C (PLC)-related catalytically inactive protein 1 (PRIP-1) in decidual cells blocks Ca^2+^ release from the ER by inhibiting inositol 1,4,5-trisphosphate (IP3) signaling [74]. Most importantly, knockout of Orai1 on the specific Institute of Cancer Research (ICR) genetic background mice did not abrogate female fertility [75]. Unlike mice lacking *Orai1* on a C57/DBA/129 background, the *Orai1*-deficient ICR mice did not suffer from high incidence of perinatal lethality [75] and may have partially replaced the function of Orai1 by other mechanisms. Further experiments are required to identify those mechanisms and explore their LEFTY2 sensitivity.

Our findings provide a novel mechanistic explanation for the clinical observation that elevated LEFTY2 levels are associated with implantation failure and infertility [13]. Taken together, our observations provide further evidence that coordinated temporal and spatial regulation of Ca^2+^ signaling in the endometrium across the window of receptivity is critical for reproductive success.

## Materials and methods

### Patient selection and sample collection

The study was approved by the NHS National Research Ethics–Hammersmith and Queen Charlotte’s & Chelsea Research Ethics Committee (1997/5065). The biopsies were timed between 6 and 10 days after the pre-ovulatory luteinizing hormone (LH) surge. None of the subjects were on hormonal treatments for at least 3 months prior to the procedure. Written informed consent was obtained from all participants in accordance with the guidelines in The Declaration of Helsinki 2000.

### Cell culture

Human endometrial stromal cells (HESCs) were isolated from endometrial tissues as described previously [76]. Purified HESCs were expanded in maintenance medium of DMEM/F-12 (Invitrogen, Schwerte, Germany) containing 10% dextran-coated charcoal-treated fetal bovine serum (DCC-FBS; Invitrogen, UK) and 1% antibiotic-antimycotic solution (Invitrogen). Confluent monolayers were decidualized in DMEM/F-12 containing 2% DCC-FBS with 0.5 mM 8-bromo-cAMP (8-Br-cAMP; Sigma, Munich, Germany) with or without 10^−6^ M medroxyprogesterone acetate (MPA; Sigma) to induce a differentiated phenotype. Where indicated, the cells were treated with recombinant LEFTY2 (25 ng/ml; R&D Systems, Germany) as described previously [77]. Ionomycin was used at 1 μM (Sigma) and the Orai inhibitors: 2-APB, YM-58483, and MRS-1845(TOCRIS, Germany). Ishikawa cells, an endometrial epithelial-like cell line (ECACC 99040201) [28, 29], were maintained in DMEM/F12 (Invitrogen) supplemented with 10% fetal bovine serum (Invitrogen), 2 mM L-glutamine, and 100 U/ml penicillin/streptomycin (Invitrogen). All cells were incubated at 37 °C in a humid atmosphere maintained at 5% (vol/vol) CO_2_, and routinely tested for mycoplasma infection.

### Animal experiments

C57BL/6 mice were purchased from Charles River Ltd. (Margate, UK). All experiments were carried out in accordance with the UK Home Office regulations (PPL70/6867). Mice had free access to food and water ad libitum*,* and were kept under constant humidity (55 ± 10%), temperature (22 ± 2 °C), and 12 h light-dark cycle conditions. To assess implantation, C57BL/6 female mice were mated by vasectomized males and the day of the appearance of the vaginal plug designated as day 1.0 dpc. Laparotomy was performed at 3.0 dpc. Both uterine horns were injected with 100 μL PBS or LEFTY2 dissolved in PBS (500 ng/ml). After 10 min, 10 cultured blastocysts (equivalent of 3.5 dpc) were transferred to a single treated uterine horn. The uteri were harvested 72 h following surgery, implantation sites counted, and tissues fixed in formalin or snap frozen for further analysis.

### Natural mating model

We conducted timed matings by placing wild-type C57BL/6 female mice with fertile wild-type males to induce pregnancy. The day when a vaginal plug was apparent was designated as 1.0 dpc, and mice were anesthetized at 3.0 dpc and subjected to laparotomy to expose the uterus [12]. We used two groups of mice: a control group flushed with PBS (Veh) and a study group flushed with LEFTY2 (as described above). The incision was then closed to allow the mice to recover. The uteri were then harvested at 9.5 dpc and the implantation sites counted.

### Western blot analysis

Whole cell protein extracts were prepared by lysing cells in RIPA buffer. Protein yield was quantified using the Bio-Rad DC protein assay kit (Bio-Rad). Equal amounts of protein were separated by 10% SDS-polyacrylamide gel electrophoresis (SDS-PAGE) before wet-transfer onto a PVDF membrane (Amersham Biosciences, UK). Nonspecific binding sites were blocked by overnight incubation with 5% nonfat dry milk in Tris-buffered saline with 1% Tween (TBS-T; 130 mmol/L NaCl, 20 mmol/L Tris, pH 7.6, and 1% Tween) as previously described [76]. The following primary antibodies were used: anti-BMP2 (*♯*sc6895, Santa Cruz Biotechnology Inc., Texas, USA), anti-WNT4 (*♯* sc376279, Santa Cruz Biotechnology Inc), anti-Cox2 (*♯*15191; Abcam, Cambridge, UK), and anti-GAPDH (*♯*21185; Cell Signaling, Leiden, The Netherlands). All primary antibodies were diluted 1:1000. Protein complexes were visualized with a chemiluminescent detection kit (WesternBright™ ECL, Advansta, CA, USA).

### Real-time quantitative (qRT)-PCR

Total RNA was extracted from cell cultures or from snap frozen whole uteri using Trizol (Invitrogen) based on a phenol-chloroform extraction protocol [78]. Equal amounts of total RNA (2 μg) were reverse transcribed using the Superscript III First-Strand synthesis system for RT-PCR (Invitrogen) with oligo dT priming. The resulting first-strand cDNA was diluted and used as a template in qRT-PCR analysis. *L19* and *Cyclophilin A* (*Cyclo*), representing non-regulated human and murine housekeeping genes, respectively, were used to normalize for variances in input cDNA. Detection of gene expression was performed with KappaFast-SYBR Green (Peqlab, Erlangen, Germany), and qRT-PCR was performed on a BioRad iCycler iQ™ Real-Time PCR Detection System (Bio-Rad Laboratories, Munich, Germany). The non-template control (NTC) reactions (cDNA was substituted with DNase/RNase free water) and reverse transcriptase (RT) controls were included in each PCR reaction. The PCR products were not detected in NTC or RT control reactions (data not shown). Transcript levels were determined by the ΔΔ_Ct_ method [79] and expressed in arbitrary units. All measurements were performed in triplicate. Melting curve analysis and agarose gel electrophoresis confirmed amplification specificity. Primer sequences are provided on request.

### Flow cytometry

Orai1 expression was analyzed by flow cytometry. Cultured cells were detached, washed three times with phosphate-buffered saline (PBS), and fixed with 4% paraformaldehyde for 15 min on ice. The cells were then incubated for 60 min (37 °C) with anti-Orai1 monoclonal primary antibody (1:200, Abcam) washed once in PBS, and stained in 1:250 diluted CF™ 488A-labeled anti-rabbit secondary antibody (Sigma) for 30 min (37 °C). Samples were immediately analyzed on a FACSCalibur flow cytometer (BD Biosciences, Heidelberg, Germany). Data were analyzed using the FlowJo software (FlowJo LLC, Ashland, Oregon, USA).

### Calcium measurements

Fura-2 fluorescence was utilized to determine [Ca^2+^]_i_ [80]. Cells were loaded with Fura-2/AM (2 μM; Invitrogen) for 20 min at 37 °C. Cells were excited alternatively at 340 and 380 nm through an objective (Fluor ×40/1.30 oil) built in an inverted phase-contrast microscope (Axiovert 100, Zeiss, Oberkochen, Germany). Emitted fluorescence intensity was recorded at 505 nm. Data were acquired using specialized computer software (Metafluor, Universal Imaging, Downingtown, USA). Cytosolic Ca^2+^ activity was estimated from the 340 nm/380 nm ratio. SOCE was determined by extracellular Ca^2+^ removal in the presence of sarcoendoplasmatic Ca^2+^ ATPase inhibitor thapsigargin (1 μM, Invitrogen) and subsequent Ca^2+^ re-addition [81]. For quantification of Ca^2+^ entry, the slope (delta ratio) and peak (delta ratio) were calculated following re-addition of Ca^2+^ [80, 82]. Experiments were performed with Ringer solution containing (in mM) 125 NaCl, 5 KCl, 1.2 MgSO_4_, 2 CaCl_2_, 2 Na_2_HPO_4_, 32 HEPES, 5 glucose, and pH 7.4. To reach nominally Ca^2+^-free conditions, experiments were performed using Ca^2+^-free Ringer solution containing (in mM) 125 NaCl, 5 KCl, 1.2 MgSO_4_, 2 Na_2_HPO_4_, 32 HEPES, 0.5 EGTA, 5 glucose, and pH 7.4. For calibration purposes, ionomycin (10 μM, Sigma) was applied at the end of each experiment.

### Histology

For histological assessment, uterine horns were formalin-fixed and embedded in paraffin, cut in 5-μm sections, and stained with H&E as previously described [12].

### Statistical analysis

Data were analyzed with the statistical package GraphPad Prism (GraphPad software Inc., CA, USA). Student’s *t* test or Kruskal-Wallis test was used when appropriate. Statistical significance was assumed when *P* < 0.05.

## Electronic supplementary material

ESM 1 (PDF 217 kb)
