# Supplementary material for: LEFTY2 inhibits endometrial receptivity by downregulating Orai1 expression and store-operated Ca2+ entry
Source: J Mol Med (Berl). 2017 Dec 11;96(2):173–82. doi: 10.1007/s00109-017-1610-9 (PMC5778154; doi:10.1007/s00109-017-1610-9)
Supplement: Supplementary file 1 — (PDF 217 kb) [file 109_2017_1610_MOESM1_ESM.pdf]

J Mol Med 2017

Electronic Supplementary Material

LEFTY2 Inhibits Endometrial Receptivity by Downregulating Orai1 Expression and Store-Operated Ca^{2+} Entry

Madhuri S Salker[1,2], Yogesh Singh[1], Ruban RP Durairaj[3], Jing Yan[1], Md Alauddin[1,2], Ni Zeng[1], Jennifer H. Steel[4,5], Shaqiu Zhang[1], Jaya Nautiyal[4], Zoe Webster[5], Sara Y Brucker[2], Diethelm Wallwiener[2], B Anne Croy[6], Jan J Brosens[3,7*] and Florian Lang[1,8*]

[1]Department of Physiology I, Eberhard-Karls University Tuebingen, Gmelinstr 5, D- 72076 Tuebingen, Germany.

[2]Department of Women's Health, Eberhard-Karls University Tuebingen, Calwerstr 7, 72076 Tuebingen, Germany.

[3]Division of Biomedical Sciences, Warwick Medical School, CV2 2DX Coventry, UK.

[4]Department of Surgery and Cancer, Institute of Reproductive and Developmental Biology, Faculty of Medicine, Imperial College London, London W12 0NN, UK

[5]ES Cell and Transgenics Facility, Medical Research Council Clinical Sciences Centre, Imperial College London, Du Cane Road, London W12 0NN, UK

[6]Department of Biomedical and Molecular Sciences, Queen's University, Kingston, Ontario, Canada

[7]Tommy's National Centre for Miscarriage Research, University Hospitals Coventry and Warwickshire NHS Trust, Clifford Bridge Rd, Coventry CV2 2DX,UK

[8]Department of Molecular Medicine II, Heinrich Heine University Düsseldorf, Universitätsstrasse 1, Düsseldorf D-40225, Germany

Correspondence to:

*Prof. Dr. Florian Lang

Physiologisches Institut der Universität Tübingen, Gmelinstr. 5, D-72076 Tübingen, Germany; email: florian.lang@uni-tuebingen.de

*Prof. Dr. Jan J Brosens

Division of Biomedical Sciences, Warwick Medical School, CV2 2DX Coventry, United Kingdom; email:J.J.Brosens@warwick.ac.uk

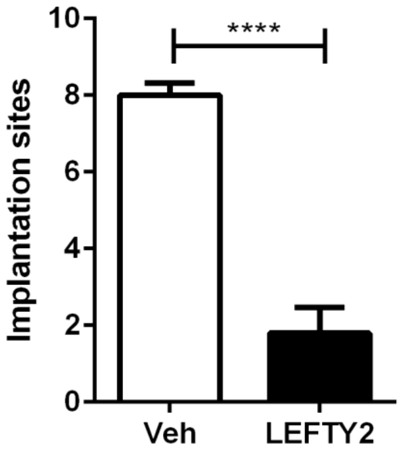

Supplementary Figure 1: LEFTY2 impairs blastocyst implantation in mice.

C57BL/6 female mice mated with fertile males were subjected to laparotomy at 3.0 dpc and both uterine horns gently flushed with either vehicle (PBS) or recombinant LEFTY2 (500 ng/ml). Each treatment group consisted of 5 mice. Bar graph shows arithmetic means ± SEM (n = 5) of the number of implantation sites following uterine flushing with PBS (Veh; White bar) or recombinant LEFTY2 (black bar). ****($P < 0.0001$) indicate significant difference from control mice (Student's *t*-test).

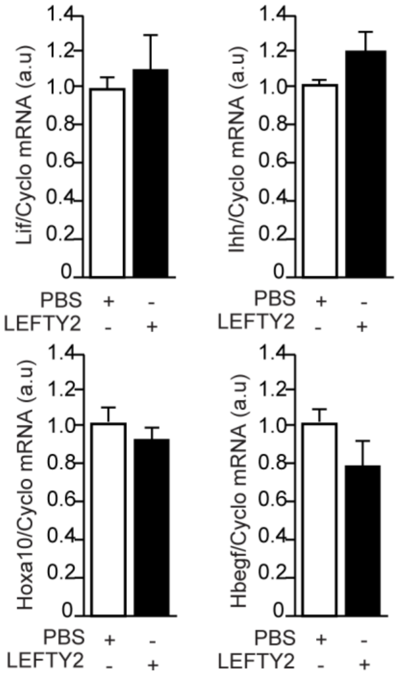

Supplementary Figure 2: Receptivity genes insensitive to LEFTY2.

Arithmetic means ± SEM (n=6) of transcript levels encoding murine *Lif, Hbegf, Hoxa10 and Ihh* levels after flushing with PBS (black bars) and with LEFTY2 (500 ng/ml; grey bars). Transcript levels were determined by qRT-PCR, normalized to the levels of *Cyclo* (housekeeping) mRNA and expressed in arbitrary units (a.u.). No significant difference was seen.

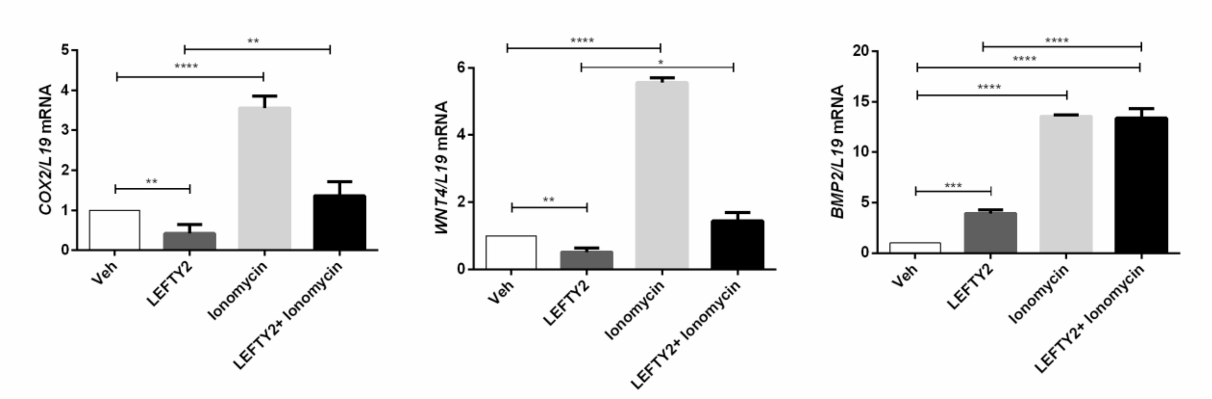

Supplementary Figure 3: Differential regulation of Ca^{2+}-dependent receptivity genes in response to LEFTY2 and Ionomycin.

Primary HESC cultures were treated with 8-Br-cAMP and MPA for 6 days with or without LEFTY2 (25 ng/ml) or Ionomycin (1 µM) or in combination. Transcript levels encoding human *COX2, WNT4* and *BMP2* were determined by qRT-PCR and normalized to *L19* (housekeeping) mRNA and expressed in arbitrary units (a.u.). The data shown are arithmetic means ± SEM (n=6). **(P<0.01) and ***(P<0.001) indicates significant difference when using Student’s *t*-test.

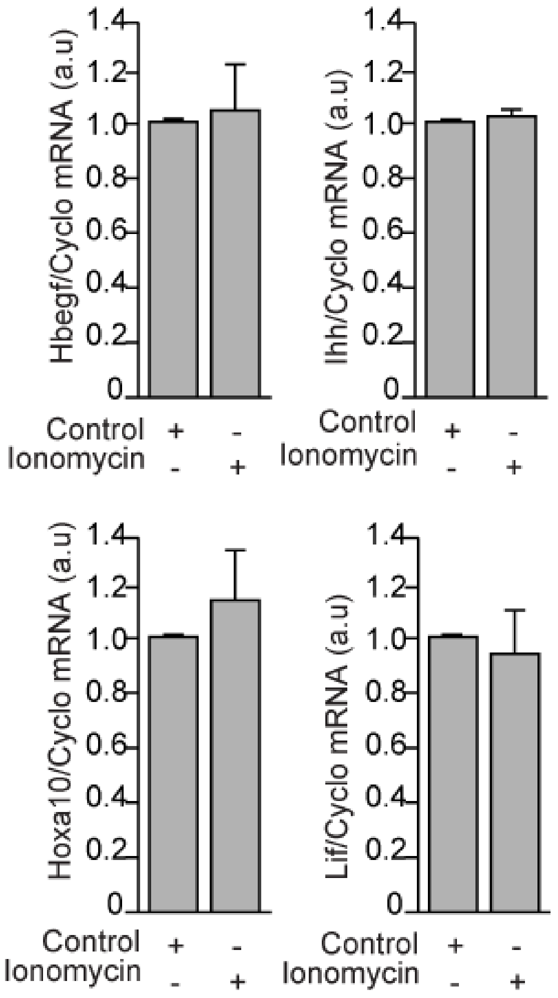

Supplementary Figure 4: Receptivity genes insensitive to Ionomycin.

Primary HESC cultures were treated with 8-Br-cAMP and MPA for 6 days with or without Ionomycin (1 µM). Arithmetic means ± SEM (n=6) of transcript levels encoding human *LIF, HBEGF, HOXA10, IHH t*ranscript levels determined by qRT-PCR, normalized to the levels of *L19* mRNA and expressed in arbitrary units (a.u.). No significant difference was seen.

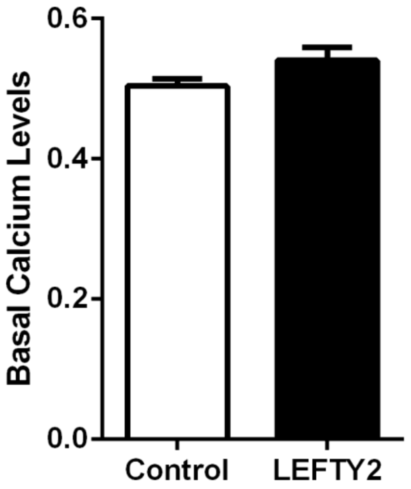

Supplementary Figure 5: Basal Calcium Levels

Arithmetic means ± SEM (n = 6, each experiment 15-30 cells) of basal Ca^{2+} of Control (Veh) and LEFTY2 treated cells. No significant difference was seen.
